# Lower Limb Kinematics in Individuals with Hip Osteoarthritis during Gait: A Focus on Adaptative Strategies and Interlimb Symmetry

**DOI:** 10.3390/bioengineering8040047

**Published:** 2021-04-13

**Authors:** Micaela Porta, Massimiliano Pau, Bruno Leban, Michela Deidda, Marco Sorrentino, Federico Arippa, Giuseppe Marongiu

**Affiliations:** 1 Department of Mechanical, Chemical and Materials Engineering, University of Cagliari, 09123 Cagliari, Italy; m.porta@dimcm.unica.it (Micaela Porta); massimiliano.pau@dimcm.unica.it (Massimiliano Pau); bruno.leban@dimcm.unica.it (B.L.); m.deidda32@tiscali.it (M.D.); msorrentino1989@gmail.com (M.S.); f.arippa@dimcm.unica.it (F.A.); 2 Orthopaedic and Trauma Clinic, Department of Surgical Sciences, University of Cagliari, 09042 Monserrato, Italy

**Keywords:** hip osteoarthritis (OA), gait, kinematics, symmetry

## Abstract

Among the functional limitations associated with hip osteoarthritis (OA), the alteration of gait capabilities represents one of the most invalidating as it may seriously compromise the quality of life of the affected individual. The use of quantitative techniques for human movement analysis has been found valuable in providing accurate and objective measures of kinematics and kinetics of gait in individuals with hip OA, but few studies have reported in-depth analyses of lower limb joint kinematics during gait and, in particular, there is a scarcity of data on interlimb symmetry. Such aspects were investigated in the present study which tested 11 individuals with hip OA (mean age 68.3 years) and 11 healthy controls age- and sex-matched, using 3D computerized gait analysis to perform point-by-point comparisons of the joint angle trends of hip, knee, and ankle. Angle-angle diagrams (cyclograms) were also built to compute several parameters (i.e., cyclogram area and orientation and Trend Symmetry) from which to assess the degree of interlimb symmetry. The results show that individuals with hip OA exhibit peculiar gait patterns characterized by severe modifications of the physiologic trend at hip level even in the unaffected limb (especially during the stance phase), as well as minor (although significant) alterations at knee and ankle level. The symmetry analysis also revealed that the effect of the disease in terms of interlimb coordination is present at knee joint as well as hip, while the ankle joint appears relatively preserved from specific negative effects from this point of view. The availability of data on such kinematic adaptations may be useful in supporting the design of specific rehabilitative strategies during both preoperative and postoperative periods.

## 1. Introduction

Hip osteoarthritis (OA) is a progressive musculoskeletal disorder that affects approximately 7 to 13% of individuals aged over 60 years old in the general population [1,2]. Hip OA manifests as a degeneration of the tissues of the joint due to different etiological factors. In particular, obesity [3,4], engaging in physically demanding jobs that require carrying heavy loads [5,6], and being a professional athlete [7] have been recognized as factors able to increase the risk of developing OA. Furthermore, genetics appear to play an important role in OA onset, since it is more prevalent in the Caucasian population with respect to Asian, Black, and East Indian populations [8].

Hip OA is associated with pain, stiffness, and functional limitation in several activities of daily living [9] including walking capabilities, thus seriously compromising the overall quality of life of the affected individual. In particular, the typical gait pattern of people with OA is characterized by reduced speed, shorter stride length, and shorter stance phase duration in the affected limb with respect to the contralateral limb. Since the success of surgical treatments and subsequent rehabilitation programs is mostly entrusted to the recovering of suitable postural control and gait efficiency, in the last decades the use of objective techniques to accurately quantify gait features has become widespread. In particular, several studies have investigated lower limb kinematics and kinetics during gait [10], thus making a considerable contribution to the assessment of outcomes associated with different surgical approaches, type of implants, and rehabilitation techniques [11].

However, despite the increasing interest in applying quantitative techniques to support clinical decisions (in terms of preoperative planning and postoperative care and rehabilitation), several aspects of gait kinematics of people with hip OA remain relatively less explored. In particular, only few studies report information on the joint kinematic alteration induced in the contralateral limb [12,13,14] and, to the best of our knowledge, no comparisons between the angle variations of hip, knee, and ankle joints of the affected and non-affected limbs have been performed on a point-by-point basis. Moreover, few data are available in terms of interlimb symmetry from the kinematic point of view [15]. Since early clinical intervention to address the deficits in gait functionality may result in improved long-term outcomes in individuals with hip OA, a more detailed understanding of biomechanical factors that influence the symmetry of lower limb joint kinematics in people with hip OA may improve management of the disease through better targeted exercise programs. Moreover, when these patients eventually receive a total hip replacement (THR), this information could be crucial for the surgeon in recreating symmetry, limb length, and conversely a “native hip”. On the basis of such considerations, the main purpose of the present study is to characterize lower limb joint kinematics during gait in individuals with hip OA using summary indexes of gait quality and symmetry indexes calculated from the angular trend associated with the entire gait cycle for each joint, and by comparing the angular trends of both the affected and unaffected limbs with respect to unaffected individuals.

## 2. Materials and Methods

### 2.1. Participants

A convenience sample of 11 individuals diagnosed with primary hip osteoarthritis (OA) currently followed at the Department of Orthopaedics of the “Nostra Signora di Bonaria” General Hospital (San Gavino Monreale, Italy) was recruited for the study on a voluntary basis. Osteoarthritis was classified according to Kellgren–Lawrence criteria [16] and all patients had severe (grade 4) coxarthritis. All participants were free from any other neurologic and orthopedic conditions able to severely affect gait and balance. They underwent a computerized three-dimensional gait analysis at the Laboratory of Biomechanics and Industrial Ergonomics of the University of Cagliari (Cagliari, Italy) prior to THR surgery. Eleven age- and sex-matched unaffected individuals, recruited among the personnel of the University of Cagliari, formed the control group (CG). The main anthropometric and clinical features of all participants are reported in Table 1.

The study was conducted according to the principles expressed in the World Medical Association Declaration of Helsinki, and all participants signed a written informed consent form with detailed information about the aims of the study.

### 2.2. Spatio-Temporal and Kinematic Data Collection and Processing

**Figure 1:**
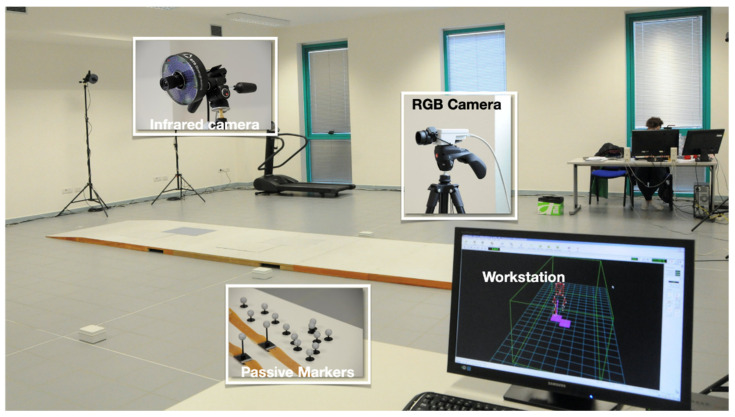
Experimental setup. The optoelectronic motion capture system is composed of 8 infrared cameras set at 120 Hz frequency, two RGB cameras for documentation purposes, and a workstation to collect, integrate and analyze data.

The kinematics and spatio-temporal parameters of gait were assessed using a motion-capture system composed of 8 infrared cameras (Smart-D, BTS Bioengineering, Italy) set at a frequency of 120 Hz (Figure 1).

**Figure 2:**
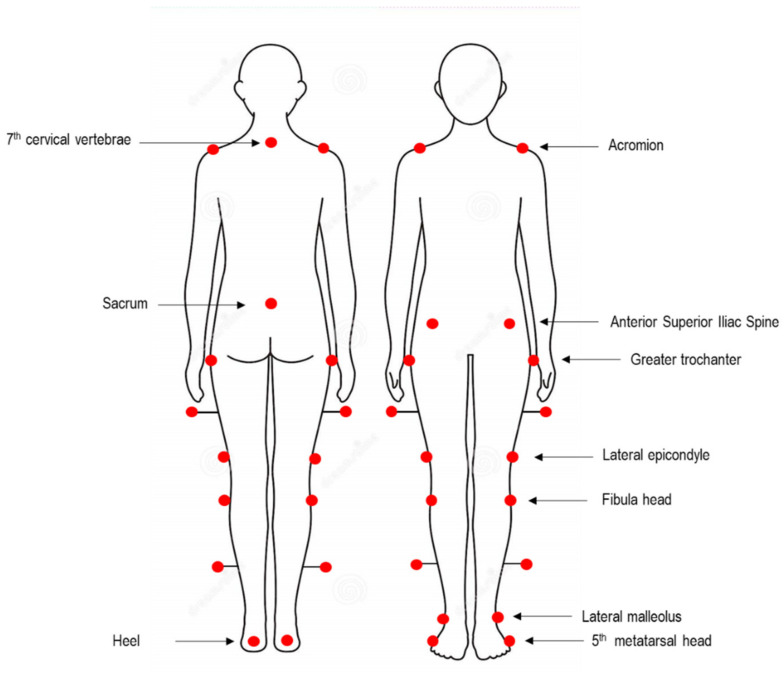
Graphic representation of markers placement according to the protocol proposed by Davis et al. [17].

Before the experimental tests, anthropometric data (i.e., height, weight, anterior superior iliac spine distance, pelvis thickness, knee and ankle width, leg length) were acquired, and 22 spherical retro-reflective passive markers were placed on subjects’ skin following the protocol described by Davis et al. [17]. In particular, three markers were used to describe the pelvic plane (i.e., right and left anterior superior iliac spine, ASIS, and the midpoint between the posterior superior iliac spine, Sacrum), eight markers for each side were used to describe the thigh and shank planes and the foot segment (i.e., greater trochanter, femoral wand, lateral epicondyle, fibula head, tibial wand, lateral malleolus, and 5th metatarsal head), and finally, four markers were used to describe the plane passing through the shoulder girdle (i.e., bilaterally on the acromion, sternum, and 7th cervical vertebrae) (Figure 2).

Lower limb kinematics were recorded while the subjects walked at a self-selected speed in the most natural manner, along a 10 m long walkway for at least 6 times, providing sufficient rest time if needed. The raw data were first processed with the dedicated Smart Analyzer (BTS Bioengineering, Italy) software to calculate:

- Five spatiotemporal gait parameters, namely gait speed, cadence, step length, step width, stance, swing, and double support phase duration;
- Nine kinematic parameters: pelvic tilt, rotation, and obliquity; hip flexion-extension, adduction-abduction, and rotation; knee flexion-extension, ankle dorsi-plantarflexion, and foot progression. From these, summary indexes of gait deviation from normality (Gait Variable Score GVS and Gait Profile Score, GPS, [18]) were obtained. GPS and GVS were previously employed to characterize gait quality in individuals who underwent THR surgery [19];
- The dynamic range of motion (ROM) for hip and knee flexion-extension and ankle dorsi-plantarflexion. They were calculated as the difference between the maximum and minimum angle value recorded during the gait cycle;
- The kinematics of hip, knee, and ankle on the sagittal plane, i.e., hip and knee flexion-extension and ankle dorsi-plantarflexion angle variations during the gait cycle. Such data were also employed to calculate the interlimb symmetry parameters as described later in detail.

### 2.3. Gait Symmetry Quantification by Means of Cyclograms

**Figure 3:**
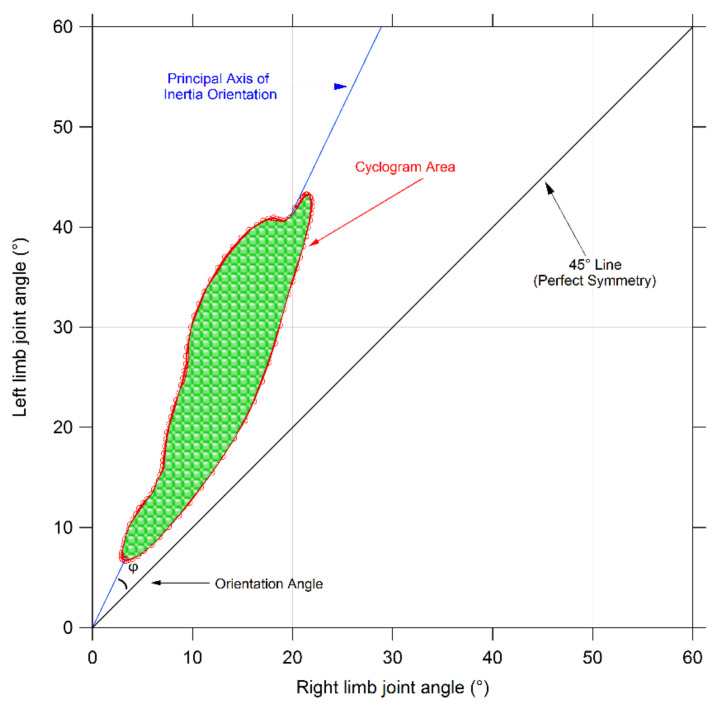
Graphic representation of a cyclogram and its main features considered for the present study.

Synchronized bilateral cyclograms were calculated using a custom routine developed under a Matlab environment following the procedure proposed by Goswami [20]. In short, right and left limb angle values acquired during the gait cycle were employed to build angle-angle diagrams for hip, knee, and ankle joints. From such diagrams, we calculated the following symmetry parameters (see Figure 3 for a graphical explanation):

- Cyclogram area (degrees^2^): the area enclosed by the curve obtained from each angle-angle diagram [21]. In the case of perfectly symmetrical gait, left and right joints assume the same angular position at a certain time, thus all cyclogram points lie on a 45° line in the diagram, making the area null. As asymmetry increases, so does the area;
- Cyclogram orientation (degrees): this parameter is expressed by the absolute value of angle ϕ formed by the 45° line (which corresponds to perfect interlimb symmetry) and the orientation of the principal axis of inertia, which corresponds to the minimum moment of inertia of the cyclogram [20,22]. The latter was calculated as the direction of the eigenvector of the matrix of inertia of the cyclogram points distribution in the x-y (i.e., left joint angle-right joint angle) reference system. Smaller values of this angle indicate higher interlimb symmetry. Higher interlimb symmetries are associated with smaller values of this angle;
- Trend Symmetry: this dimensionless parameter is calculated to assess the similarity between two waveforms (in our case time-normalized right leg and left leg angular trend across the gait cycles for each joint of interest) using an eigenvector analysis (see [23] for details of the mathematical procedure as proposed by Crenshaw et al.). In particular, it is obtained by dividing the variability about the eigenvector to the variability along the eigenvector. Trend Symmetry is not affected by the presence of a shift or by magnitude differences in the two waveforms. A null value indicates perfect symmetry, while increasing interlimb asymmetry corresponds to higher Trend Symmetry values.

### 2.4. Statistical Analysis

A statistical analysis was conducted to evaluate the different gait parameters considered as follows:

- Spatio-temporal parameters were analyzed after separating them into two groups: (i) gait speed, cadence, and step width (for which both limbs are involved) and (ii) stance, swing, double support phases, and step length, which can be analyzed distinguishing between limbs. The possible differences introduced by the presence of OA in spatiotemporal parameters were explored using a one-way multivariate analysis of variance (MANOVA) considering group (OA/CG) as the independent variable and the 3 parameters previously listed as dependent variables. In contrast, we ran a one-way MANOVA for the phase duration parameters considering the limb (affected, unaffected and CG) as independent variables and the 4 parameters previously mentioned as dependent variables.
- For dynamic ROM, GVS, and GPS analysis, two separate MANOVAs were carried out considering the limb (affected, unaffected, and CG) as the independent variable and the three ROM or the 9 GVS and GPS as dependent variables.
- The possible differences in joint kinematics were assessed by comparing the “angle vs. time” curves of the affected limb, the unaffected limb, and CG on a point-by-point basis using a one-way ANOVA for each of the 3 joints of interest, similarly to what was proposed by Bruening et al. [24]. It was thus possible to define the periods of the gait cycle in which significant differences associated with participants’ limb were present.
- Finally, possible differences introduced by OA in gait symmetry parameters were investigated using a MANOVA, considering group (OA/CG) as the independent variable and the 3 symmetry parameters previously listed as dependent variables.

To compare gait parameters related to the affected and unaffected limbs with respect to CG, a preliminary analysis was performed to exclude the existence of significant differences in the investigated parameters between CG’s left and right limbs. Since such differences were not found, the mean value of each investigated parameter calculated across the two limbs was considered representative of each participant enrolled in the CG.

In all cases, the level of significance was set at *p* = 0.05 and the effect sizes were assessed using the eta-squared (η^2^) coefficient. For each MANOVA (i.e., cases a, b, and d), univariate ANOVA was performed as a post-hoc test by reducing the level of significance to *p* = 0.05/n (where n is the number of the dependent variables) after a Bonferroni adjustment for multiple comparisons. Where necessary, post-hoc Holm–Sidak tests were performed to assess pairwise intra- and inter-group differences. All analyses were performed using the SPSS version 20b software (IBM SPSS Statistics, Armonk, NY, USA).

## 3. Results

**Figure 4:**
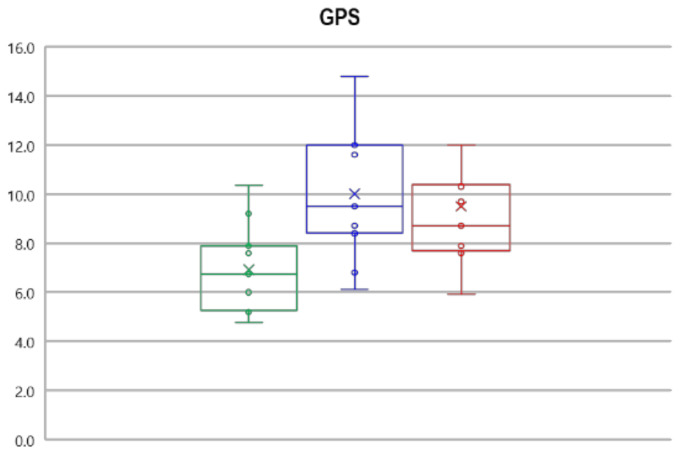
Box plot summarizing the values of GPS for each group considered. The green line represents the control group, the blue line represents the unaffected limb of individuals with OA, and the red line represents the affected limb of individuals with OA.

**Figure 5:**
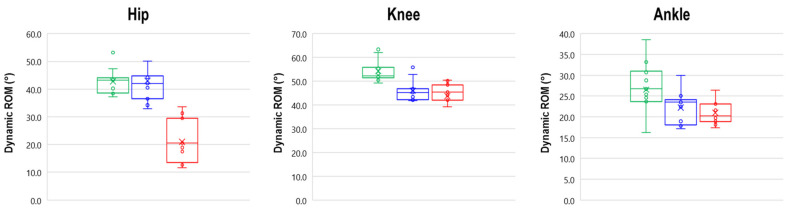
Box plots summarizing the dynamic ROM for hip, knee, and ankle joints. The green line represents the control group, the blue line represents the unaffected limb of individuals with OA, and the red line represents the affected limb of individuals with OA.

**Figure 6:**
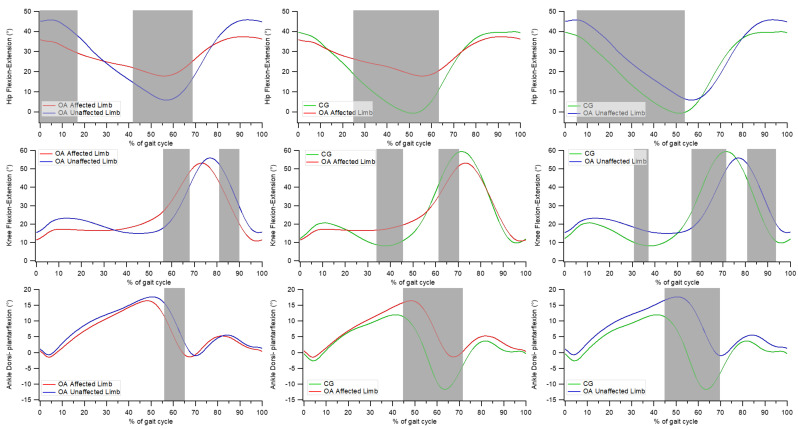
Gait kinematics in the sagittal plane. From top to bottom: hip flexion-extension, knee flexion-extension, and ankle dorsi-plantar-flexion angles during gait cycle. From left to right: comparison between the affected and unaffected limbs in people with OA, comparison between the affected limb of people with OA and healthy controls, comparison between the unaffected limb of people with OA and healthy controls. The grey-shaded areas denote the periods of the gait cycle in which a significant difference between the groups existed (*p* < 0.05).

**Figure 7:**
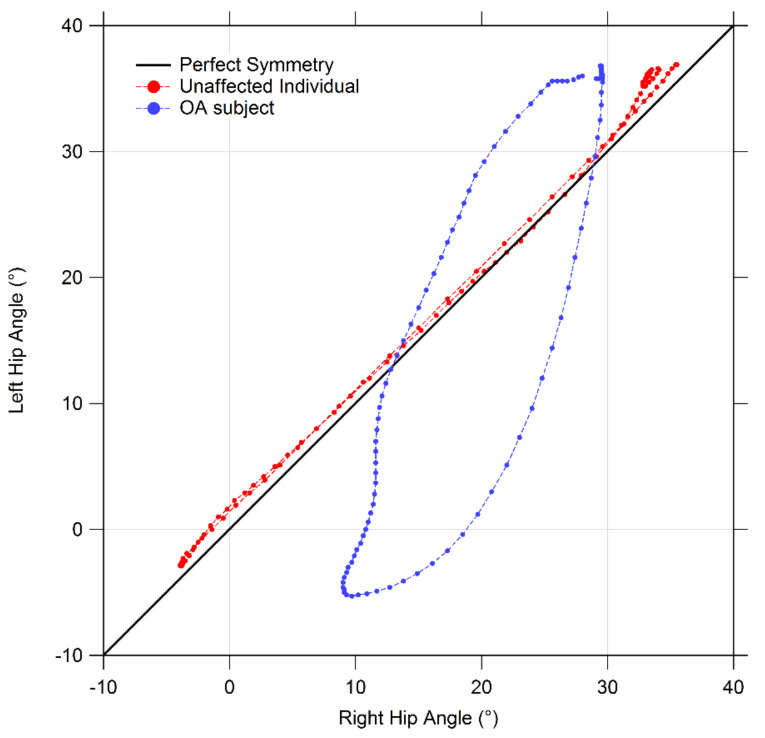
Example of cyclograms for individuals with hip OA and healthy controls.

**Figure 8:**
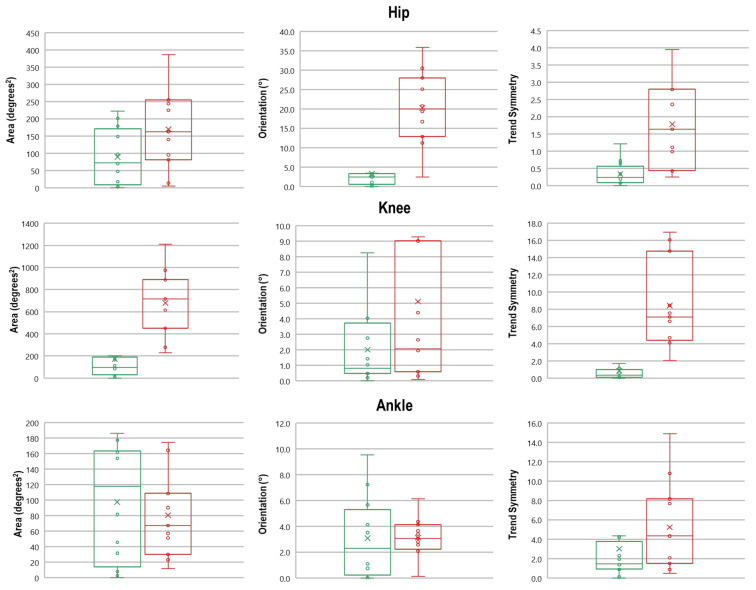
Box plots summarizing Area, Orientation, and Trend Symmetry for hip, knee, and ankle joints. The green line represents the control group and the red line individuals with OA.

The results of the comparison between individuals with OA and unaffected individuals with regard to the spatial-temporal parameters GVS, GPS, dynamic ROM of gait, and symmetry indexes are summarized in Tables 2–5. Graphical representations of the comparison between individuals with OA and unaffected individuals of GPS values, dynamic ROM, and symmetry index together with the angle variations in the sagittal plane during the gait cycle for hip, knee, and ankle joints are reported in Figures 4–8.

### 3.1. Spatio-Temporal Parameters of Gait

MANOVA detected a significant effect of OA on the first group of spatio-temporal parameters of gait (i.e., speed, cadence and step width) (F(3,18) = 17.257, *p* < 0.001, Wilks λ = 0.258, η^2^ = 0.742). The subsequent follow-up ANOVA indicated that individuals with OA are characterized by significantly reduced velocity and cadence (*p* < 0.001). A significant main effect of the disease was also detected when analyzing stance phase, swing phase, and step length (F(8,54) = 8.205, *p* < 0.001, Wilks λ = 0.204, η^2^ = 0.582). In particular, the follow-up ANOVA indicated that individuals with OA exhibited a prolonged stance phase for both the affected and unaffected limbs compared with CG (*p* < 0.001 unaffected vs. CG, *p* = 0.003 affected vs. CG), a reduced swing phase of the unaffected limb (*p* < 0.001), and a prolonged double support phase duration for both the affected and unaffected limbs compared to CG (*p* = 0.017 and *p* = 0.006 respectively). Finally, step length was also found to be significantly reduced in both the affected and unaffected limbs with respect to CG (*p* < 0.001) (Table 2).

### 3.2. Gait Kinematics, GPS and GVS

The statistical analysis revealed a significant main effect of limb on GPS and GVS indexes (F(10,42) = 3.087, *p* = 0.001, Wilks λ = 0.164, η^2^ = 0.595). The subsequent post-hoc analysis showed that individuals with OA exhibited increased GPS for both the affected and unaffected limbs in comparison to CG and increased GVS for pelvic obliquity and hip flex-extension. A significant increase in GVS was also found for the pelvic rotation, hip ab-adduction, and ankle dorsi-plantarflexion of the unaffected limb (Table 3). Figure 4 summarizes the values of GPS for each tested group.

### 3.3. Dynamic ROM

In this case, MANOVA detected a significant main effect of limb on dynamic ROM (F(3,56) = 11.871, *p* < 0.001, Wilks λ = 0.194, η^2^ = 0.560). The subsequent follow-up ANOVA indicated that individuals with OA were characterized by significantly reduced dynamic hip, knee, and ankle ROM of the affected limb with respect to CG. Moreover, hip ROM of the affected limb was also reduced when compared to the contralateral hip, while the ROM of the contralateral knee showed a significant reduction with respect to the CG (Table 4, Figure 5).

### 3.4. Point-by-Point Analysis of Kinematic Curves

The analysis of hip, knee, and ankle kinematics in the sagittal plane (Figure 6) revealed the existence of:

- significant differences between the affected and unaffected limbs in individuals with OA at the hip joint from 0 to 17% and from 42 to 68% of the gait cycle, at the knee joint between 56 to 67% and between 81 and 90% of the gait cycle, and at the ankle joint from 56 to 65% of the gait cycle;
- significant differences between the affected limb of individuals with OA and CG at the hip joint from 25 to 64% of the gait cycle, at the knee joint between 34 and 46% and between 62 and 70% of the gait cycle, and at the ankle joint from 45 to 71% of the gait cycle;
- significant differences between the unaffected limb of individuals with OA and CG at the hip joint between 5 and 54% of the gait cycle; at the knee joint between 31–37%, 56–72%, and 81–94% of the gait cycle; and at the ankle joint between 45 and 70% of the gait cycle.

### 3.5. Symmetry Indexes

MANOVA detected a significant effect originated by the presence of OA on symmetry indexes at hip and knee joints (hip F(3,18) = 22.165, *p* < 0.001, Wilks λ = 0.213, η^2^ = 0.787, knee F(3,18) = 3.087, *p* = 0.001, Wilks λ = 0.414, η^2^ = 0.586) but not at the ankle joint. In particular, the post-hoc analysis revealed that the cyclogram area was significantly larger in individuals with OA only at the knee joint. In contrast, both cyclogram orientation and Trend Symmetry parameters at the hip joint were found significantly higher in people with OA. Higher Trend Symmetry in people with OA with respect to CG was also detected at the knee joint (see Table 5). Figure 7 reports an example of cyclograms obtained for individuals with hip OA and healthy controls. Figure 8 reports the box plots which summarize the symmetry parameters for hip, knee, and ankle joints in both groups.

## 4. Discussion

The main aim of the present study was to characterize the main alterations in gait kinematics in individuals affected by hip OA, with a special focus on interlimb asymmetry and detailed point-by-point comparison of the angular trends of hip, knee, and ankle joints between the affected and unaffected limbs of people with OA and with respect to CG. 

Firstly, it is noteworthy that our results on spatio-temporal parameters are substantially consistent with most previous studies since the gait of individuals with hip OA was found to be characterized by reduced speed, stride length, cadence, and swing phase duration and increased stance and double support phase duration [10,25,26]. We also detected significant differences in stance and swing phase duration between the affected and unaffected limbs of people with OA, which indicates the existence of a specific antalgic strategy aimed at reducing forces applied on the affected joint [12].

The analysis of the quality of gait from a kinematic point of view, performed calculating the summary indexes GPS and GVS, showed a variety of alterations in both limbs which are particularly relevant with regard to hip flexion-extension (with a GVS value almost double with respect to CG), ankle dorsi-plantar-flexion, and, to a lesser extent, knee flexion-extension. A relevant deviation from physiological gait was also found in pelvic tilt, rotation, and obliquity. Differences in pelvic tilt may be associated with increased lumbar flexion extension, which has been identified as a method of compensation for the limited range of motion of the hip during gait [27]. The overall values calculated here for the GPS (9.5° and 10° for the affected and unaffected limbs, respectively) were found in good agreement with those recently reported by Temporiti et al. [19]. Taken together, these anomalies suggest that the presence of hip OA may also influence mobility of the other joints of both ipsi- and contralateral limbs. 

As previously mentioned, one of the novelties of this study is represented by the point-by-point analysis carried out to explore the differences between joint kinematic curves that evolve during the gait cycle. Such an approach allows a better understanding of where gait alterations of the cycle are located, thus providing useful insights mainly in terms of pre- and postoperative exercise training. Firstly, when considering the affected vs. unaffected limb comparison, we observe that at hip level, the differences involve the heel contact and terminal stance/initial swing phases, with the affected hip that exhibits a deficit of flexion in the former period and a deficit of extension in the latter, consistent with what was previously reported by Ismailidis et al. [14] and Zeni et al. [28]. It has been suggested that such a phenomenon might be due to an increase in pelvis and/or trunk flexion developed as a strategy to avoid pain [10].

In contrast, few differences were detected at the knee and ankle joints. In particular, we observed significant changes between the affected and unaffected limbs only during two small periods (located at terminal stance and mid-swing), likely due to a temporal forward shift of the affected limb curve originated by the reduced duration of the stance phase (62.5% vs. 67.4%). The same cause probably explains the small differences present in the ankle curves at the terminal stance. 

The comparison between the kinematic curves that refer to the unaffected limb of people with OA and CG revealed the existence of several relevant alterations, probably caused by adaptation strategies created by the presence of the disease. At the hip joint, most of the differences involve the stance phase of the gait cycle, during which people with OA systematically exhibit higher values of flexion. Besides, hip kinematics shows a “hesitation” in flexion-extension motion during stance with respect to the CG. 

Such findings suggest that people with OA who experience pain, hip flexor contractures, and muscle weakness [27] tend to adopt specific strategies to decrease hip joint load, thereby alleviating pain on an instable and worn-out joint. Moreover, higher values of hip flexion have been to be found associated with a leaned trunk posture as well as a limited dynamic hip range of motion. As regards the knee joint, it is noteworthy that there is a certain lack of extension through most of the stance phase (even though statistically significant only in mid stance) and at the terminal stance, while it becomes larger with respect to unaffected individuals in the mid-swing, again probably due to a temporal mismatch of the cycle caused by different stance phase duration. Finally, important alterations were also found at ankle level, especially during terminal stance, mostly expressed as a marked lack of plantar flexion.

The analysis of interlimb joint kinematics symmetry, which was performed using waveform-based methods, shows that individuals with OA exhibit a marked asymmetry at hip and knee joints, but not at the ankle. While the existence of asymmetry at the hip joint is negligible, it is interesting to note the extent of this phenomenon. In fact, both orientation and Trend Symmetry values were found to be 5–6 times higher than those of CG. Just to provide a term of comparison, Goswami et al. [20] reported increases of 2 to 3 times for cyclogram orientation values at the hip joint in a small sample of people with stroke when comparing the paretic and non-paretic limb. Similarly, even at knee level, both cyclogram area and Trend Symmetry were significantly higher with respect to those of CG, thus indicating that the negative influence associated with reduced efficiency of the affected hip extends also to more distal joints. However, such an effect appears to vanish at ankle level as the symmetry parameters of people with OA were not found significantly different from those of CG. 

Some limitations of the research are to be acknowledged. Firstly, the limited size of the sample tested here suggests caution when generalizing the result, despite the medium-to-large effect size values obtained from the statistical analysis. The same factor forced us to pool participants of both sexes into a single group, even though previous studies report that gait alterations associated with hip OA differ in men and women with hip OA [29], thus participants should be separately assessed. Moreover, we collected no information on participants’ physical fitness at the time of the experimental trials, and this is another factor known to have some influence on gait patterns of individuals with hip OA [30].

## 5. Conclusions

In this study we investigated gait patterns of individuals with hip OA, with a special focus on lower limb joint kinematics. This was done by comparing the joint angle curves of the affected and unaffected limbs and with respect to CG, and by calculating symmetry parameters derived from a waveform-based approach that considers whole curves instead of single discrete parameters. The results show that individuals with hip OA exhibit specific gait patterns which, besides the well-known alterations of spatio-temporal parameters, include severe modifications of the physiologic trend at the hip level even in the unaffected limb (especially during the stance phase), and minor (although significant) issues at knee and ankle level. The symmetry analysis also revealed that the effect of the disease on interlimb coordination is present at the knee joint as well as at the hip, while the ankle joint appears relatively exempt from specific negative effects from this point of view. What is the possible role of such analysis from a clinical point of view? Several recent studies pointed out the difficulties encountered in defining the effectiveness of physical therapy carried out before the surgery on gait performance [31]. On the other hand, other research highlighted the importance of the quantitative techniques for human movement analysis to improve the evaluation of the rehabilitation process after surgery [32]. We believe that the availability of a dataset that encompasses a wide range of gait aspects, including spatio-temporal parameters, kinematic adaptations, and symmetry, might prove useful in supporting the design of rehabilitative strategies specifically tailored to the patient’s need during both preoperative and postoperative phases, with the final aim of accelerating the recovery, improving quality of life, and reducing the overall costs for the health systems.

## Figures and Tables

**Table 1:** Demographic and anthropometric characteristics of participants. Values are expressed as mean ± SD.

	OA Group (5F, 6M)	Control Group (5F, 6M)
Age (years)	68.3 ± 5.8	67.8 ± 5.4
Body mass (kg)	75.4 ± 16.9	75.9 ± 10.1
Height (cm)	162.0 ± 5.4	165.9 ± 7.6

**Table 2:** Spatio-temporal parameters of gait. Stance, swing, and double support phases are expressed as percentage of the gait cycle duration. Values are expressed as mean ± SD.

	**Control Group**	**OA** **Unaffected Limb**	**OA** **Affected Limb**
Stance Phase	60.17 ± 2.57	67.39 ± 2.62 *	62.50 ± 4.83 ^†^
Swing Phase	39.08 ± 1.95	32.63 ± 2.61 *	36.03 ± 4.70 *^,†^
Double Support Phase	11.10 ± 1.99	17.07 ± 7.4 *	16.22 ± 2.90 *
Step Length (m)	0.60 ± 0.06	0.40 ± 0.07 *	0.40 ± 0.06 *
	**Control Group**	**OA Group**	
Mean Velocity (m/s)	1.05 ± 0.19	0.56 ± 0.12 *	
Cadence (step/min)	105.94 ± 11.12	84.69 ± 12.00 *	
Step Width (m)	0.21 ± 0.02	0.24 ± 0.03	

The symbol * denotes a significant difference with respect to the Control Group; the symbol † denotes a significant difference with respect to the unaffected limb.

**Table 3:** GPS and GVS indexes (in degrees). Values are expressed as mean ± SD.

Parameter	Control Group	OA Unaffected Limb	OA Affected Limb
GPS	6.9 ± 1.7	10.0 ± 2.6 *	9.5 ± 2.6 *
Pelvic Obliquity GVS	2.2 ± 0.6	4.2 ± 1.5 *	3.8 ± 1.0 *
Pelvic Tilt GVS	4.7 ± 3.0	9.3 ± 6.6	8.6 ± 6.2
Pelvic Rotation GVS	3.7 ± 1.2	6.0 ± 2.3 *	5.3 ± 1.6
Hip Ab-Add GVS	3.7 ± 1.0	6.1 ± 2.3*	4.1 ± 1.4
Hip Flex-Ext GVS	7.7 ± 2.9	13.4 ± 7.0	15.5 ± 6.9 *
Hip Rotation GVS	9.8 ± 3.3	9.5 ± 4.8	7.5 ± 3.0
Knee Flex-Ext GVS	9.0 ± 3.4	12.9 ± 3.0	11.6 ± 4.6
Ankle Dorsi-Plantarflex GVS	5.6 ± 1.4	10.5 ± 3.9 *	8.9 ± 3.6
Foot Progression GVS	8.1 ± 4.0	7.9 ± 4.2	9.0 ± 3.8

The symbol * denotes a significant difference with respect to the control group.

**Table 4:** Dynamic Range of Motion during gait (in degrees). Values are expressed as mean ± SD.

	Control Group	OA Unaffected Limb	OA Affected Limb
Hip	42.8 ± 4.6	43.0 ± 8.4	20.9 ± 7.5 *^,†^
Knee	54.2 ± 4.6	48.0 ± 7.1 *	43.7 ± 7.2 *
Ankle	26.5 ± 7.3	22.2 ± 3.9	21.0 ± 2.7 *

The symbol * denotes a significant difference with respect to the control group; the symbol † denotes a significant difference with respect to the unaffected limb.

**Table 5:** Comparison between symmetry indexes of people with hip OA and unaffected individuals. Values are expressed as mean ± SD.

	Cyclogram Parameter	Control Group	OA Group
Hip	Area	96.87 ± 79.6	169.60 ± 117.14
	Orientation ϕ	3.53 ±3.95	20.25 ± 9.49 *
	Trend Symmetry	0.37 ± 0.35	1.78 ± 1.24 *
Knee	Area	188.38 ± 232.21	677.38 ± 304.48 *
	Orientation ϕ	2.17 ± 2.48	5.11 ± 7.02
	Trend Symmetry	0.90 ± 1.33	8.42 ± 5.16 *
Ankle	Area	106.16 ± 72.88	80.46 ± 54.51
	Orientation ϕ	3.36 ± 3.16	3.13 ± 1.52
	Trend Symmetry	3.30 ± 4.86	5.22 ± 4.64

The symbol * denotes a significant difference with respect to the control group.

## Data Availability

The data that support the findings of this study are available from the corresponding author G.M., upon reasonable request.
